# Clinical Characteristics and Factors Associated With Mortality in First-Episode Infective Endocarditis Among Persons Who Inject Drugs

**DOI:** 10.1001/jamanetworkopen.2018.5220

**Published:** 2018-11-21

**Authors:** Laura Rodger, Stephannie Dresden Glockler-Lauf, Esfandiar Shojaei, Adeel Sherazi, Brian Hallam, Sharon Koivu, Kaveri Gupta, Seyed M. Hosseini-Moghaddam, Michael Silverman

**Affiliations:** 1 Schulich School of Medicine and Dentistry, London, Ontario, Canada; 2 Western University, London, Ontario, Canada; 3 Infectious Diseases, St Joseph's Health Care, London Health Sciences Centre, London, Ontario, Canada

## Abstract

**Importance:**

Persons who inject drugs (PWID) represent a distinct demographic of patients with infective endocarditis. Many centers do not perform valvular surgery on these patients owing to concerns about poor outcomes. Addiction services are underused in hospitals.

**Objectives:**

To compare clinical characteristics in first-episode infective endocarditis in PWID who are surgically vs medically managed and to identify variables associated with mortality.

**Design, Setting, and Participants:**

This case series studied PWID treated for a first episode of infective endocarditis between April 1, 2007, and March 30, 2016. Participants were adult patients (aged ≥18 years) admitted to any of 3 hospitals in London, Ontario, Canada. Analysis occurred between July 2016 and November 2017.

**Main Outcomes and Measures:**

Survival among PWID; the causative organisms, site of infection, and cardiac as well as noncardiac complications; referral to addiction services; and surgical vs medical management.

**Results:**

Of 370 total first-episode cases of infective endocarditis, 202 (54.6%) were in PWID. Among PWID, 105 (52%) were male, the median (interquartile range) age was 34 (28-42) years, and patients were predominantly positive for the hepatitis C virus (69.8% [141 of 202]). Right-sided infection was more common (61.4% [124 of 202]), and most infections were caused by *Staphylococcus aureus* (77.2% [156 of 202]). Surgery occurred in 19.3% of patients (39 of 202). The all-cause mortality rate was 33.7% (68 of 202). Adjusting for age and sex, survival analysis demonstrated that surgery was associated with lower mortality (hazard ratio [HR], 0.44; 95% CI, 0.23-0.84; *P* = .01), as was referral to addiction treatment (HR, 0.29; 95% CI, 0.12-0.73; *P* = .008). Higher mortality was associated with left-sided infection (HR, 3.26; 95% CI, 1.82-5.84; *P* < .001) and bilateral involvement (HR, 4.51; 95% CI, 2.01-10.1; *P* < .001).

**Conclusions and Relevance:**

This study presents the demographic characteristics of first-episode infective endocarditis in PWID. Results highlight the potentially important role of addictions treatment in this population. Further study to optimize selection criteria for surgery in PWID is warranted.

## Key Points

**Question:**

In first-episode infective endocarditis in persons who inject drugs, what are the clinical differences between patients who receive surgery vs those who are medically treated, and which factors are associated with mortality?

**Findings:**

In this case series of 370 first-episode cases of infective endocarditis, the main significant differences between persons who inject drugs who received surgery and those who did not were the site of infection and cardiac complications. Decreased mortality was associated with surgery and referral to addiction treatment services, while higher mortality was associated with left-sided and bilateral infections.

**Meaning:**

In selected persons who inject drugs with first-episode endocarditis, surgical management and referral to addiction treatment were associated with reduced mortality.

## Teaser

This case series study compares outcomes for persons in Canada who inject drugs with a first episode of infective endocarditis treated surgically vs those not treated surgically and examines factors associated with mortality.

## Introduction

Infective endocarditis (IE) refers specifically to an infection of the endocardium and heart valves or of a prosthetic valvular implant.^1^ There has been a substantial shift in the demographics of patients with IE, particularly in the developed world.^2^ It remains a significant cause of morbidity and mortality,^3,4,5^ and although the overall incidence has remained stable,^6^ the incidence has been increasing in persons who inject drugs (PWID).^7,8,9^ Among non–drug users, increasing age is associated with degenerative valvular disease; additionally, health care–associated cases are more frequent and are attributed to procedures, indwelling lines, or intracardiac devices.^2,6,10^ Conversely, among PWID, susceptibility to infection is poorly understood but hypothesized to result primarily from endothelial damage from particulate material and repeated high-grade bacteremia introduced by direct inoculation.^11,12^

Unfortunately, contemporary literature is limited when considering IE in drug users. Focused prospective data are difficult to collect, and in large cohort studies the subgroups of PWID have limited sample sizes. As a result, there continue to be discrepancies in the characterization of infections among drug users, and it is challenging to assess optimal treatment strategies. It is also important to characterize the presentation of IE within the context of the recent opioid epidemic. Therefore, we sought to better characterize PWID hospitalized with a first episode of IE, identify factors associated with mortality, and clarify the association of surgical management with mortality in PWID.

## Methods

### Population

This case series included patients admitted to any of the 3 acute care hospitals in London, Ontario, Canada (catchment area, 1.5-2 million persons). Reporting of all aspects of this study adhered to the Strengthening the Reporting of Observational Studies in Epidemiology (STROBE) reporting guideline for cohort studies.^13^ All patients were adult (aged ≥18 years) inpatients admitted between April 1, 2007, and March 30, 2016. Analysis occurred between July 2016 and November 2017. The last follow-up time was November 2017 (to ensure 12 months of follow-up after enrollment). The study population was generated by extracting all cases with a discharge diagnosis of infective endocarditis; medical records were reviewed by infectious diseases physicians using a standard form to abstract data that were then entered into a database. Only patients with definite IE per the modified Duke criteria^14^ were enrolled; these criteria have been demonstrated to accurately classify IE among PWID.^15^ The advanced electronic medical record system in London is an integrated database of clinical data (bloodwork, diagnostic imaging, microbiological studies, and clinical notes) from any health care point of contact in the city. This allowed comprehensive long-term follow-up information to be obtained. Medical record review was carried out as per a quality improvement initiative for the care of patients with endocarditis. All patient data were anonymized prior to analysis. Therefore, individual patient consent was not required. Ethical approval for the study was obtained from the Lawson Research Institute review board.

### Outcomes and Explanatory Variables

Definite IE cases were categorized as first-episode or recurrent IE, and patients were classified as PWID based on a history in the clinical record of self-reported injection drug use. Use of opioid substitution therapy (OST) at discharge and referral to addiction services were also documented. Demographic information collected for each patient included age, sex, comorbid conditions (eg, HIV or hepatitis C infection), and predisposing conditions (eg, heart disease, chronic venous access, intracardiac devices, or prosthetic valve). Health care–associated infection was defined as in previous literature.^16^ Microbiological data from blood cultures and echocardiographic data were also recorded. Important sequelae noted included cardiac complications (eg, congestive heart failure, myocardial or aortic root abscess, conduction delay, stroke, mycotic aneurysm, or septic emboli) and invasive infections (eg, central nervous system infection, osteomyelitis, or septic arthritis). Myocardial or aortic root abscess was identified by transesophageal echocardiography or computed tomography. Stroke was defined as presentation with a stroke-like syndrome with positive imaging (computed tomography or magnetic resonance imaging). Septic arthritis was defined as either (1) a positive culture from a joint or (2) positive blood cultures with a synovial fluid aspirate that was grossly purulent or with a white blood cell count of greater than 50 000/L.^17^ Treatment data included antibiotic administration, peripherally inserted central catheter (PICC) line insertion, documented or suspected PICC line misuse (patient use of PICC line to administer drugs other than antibiotics based on written opinion of the attending medical team in the medical record), surgical treatment, and intensive care unit (ICU) admission. Admission to the ICU was defined as preoperative admission, whereas postoperative recovery in the ICU was not considered. For patients who underwent surgery, the date of surgery and procedure were recorded. Death was documented from the electronic medical record, and patients were considered alive if the medical record demonstrated them to be so. Survival was followed electronically, as any patient presenting to any acute care, rehabilitation, or psychiatric (addiction) facility in the city and then dying would be captured even outside of the hospital records as any laboratory testing, radiology, or filling of a prescription in the community is noted on the comprehensive record and demonstrates that the patient remained alive. If there was no further involvement with the health care system, follow-up was considered terminated at the time of the last interaction.

Recurrent IE was defined as a new episode meeting the modified Duke criteria for definite IE based on visualization of a new vegetation on echocardiography and positive blood cultures occurring more than 6 months from a previous episode. If presenting within 6 months after the first episode, cases were described as recurrent IE only if both a new vegetation was identified and a new organism was isolated from blood cultures. If the same organism was identified, this was considered relapse of the original infection. Right-sided IE was defined as infection involving only right heart structures; left-sided IE referred to cases in which infection was localized to the left side of the heart only. Bilateral infection included cases where infection occurred on right and left structures.

### Statistical Analysis

Overall, missing data were labeled as such, and binary outcomes were coded as positive (1) or negative (0). Certain variables had a significant amount of missing data. These variables were not used for advanced analysis. When the rate of missing data was less than 10%, imputation was used under the assumption that data were missing at random. Categorical variables are presented as frequencies and percentages. Continuous variables are presented as median with interquartile range. Comparisons were made between proportions using χ^2^ tests, Fisher exact tests, and post hoc analysis, as appropriate. Continuous variables were assessed for normality and analyzed using Wilcoxon tests or *t* tests, as necessary. A multivariate Cox proportional hazards model was generated for PWID using statistically significant covariates from prefiltering univariate analysis (*P* < .20), which included age, sex, leaving against medical advice (AMA), site of infection, and referral to addiction treatment. In addition, surgery, the causative organism, and opiate substitution therapy were included initially as clinically relevant variables. Covariates were assessed for violation of the proportional hazards assumption and assessed using log-negative-log survival plots and Schoenfeld residual plots. Stepwise regression, both forward and backward selection, was used to choose the best model by Akaike information criterion. The model with the minimum Akaike information criterion value was used as the final model. Subsequent sensitivity analysis assessed the primary covariate of interest (surgery) with alternate models. Hazard ratios (HRs) and 95% confidence intervals were calculated and reported where applicable. Statistical analyses were performed with R statistical software version 1.0.143 (R Project for Statistical Computing). All tests were 2-tailed with *P* < .05 considered statistically significant.

## Results

At the time of discharge, 1464 episodes of infective endocarditis were identified in patients aged 18 years or older. Of these, 497 episodes fulfilled modified Duke criteria for definite IE. Of these episodes, a further 121 cases were excluded based on a history of previous IE. In total, 370 patients with first-episode IE were identified, with 202 (54.6%) of them having a documented history of self-reported injection drug use. These patients were included in the full study cohort. Patients were 52% male (105 of 202) with a median (interquartile range) age of 34 (28-42) years. Baseline characteristics are presented in Table 1. The median (interquartile range) duration of follow-up was 3.56 (2.27-5.75) years (95% CI, 3.24-3.99). All patients had a minimum of 1 year of follow-up. The majority of patients were positive for the hepatitis C virus antibody (69.8% [141 of 202]). Homelessness occurred in 17% of PWID (35 of 202), where no fixed address was identified. Very few patients had preexisting cardiac conditions, and there were no cases of health care–associated IE. The majority of PWID had right-sided infection (61.4% [124 of 202]) and 118 cases exclusively involved the tricuspid valve. *Staphylococcus aureus* infections were the causative organism in 77.2% of cases in PWID (156 of 202), followed by 6.4% (13 of 202) with a polymicrobial infection, and 5.4% (11 of 202) caused by enterococci.

**Table 1.** Baseline Characteristics in First-Episode Endocarditis in 202 Persons Who Inject Drugs

Variable	No./Total No. (%)
Age, median (IQR), y	34 (28-42)
Sex	
Male	105/202 (52.0)
Female	97/202 (48.0)
HIV status	
Positive	16/202 (7.9)
Negative	141/202 (69.8)
Unknown	45/202 (22.3)
Hepatitis C status	
Positive	141/202 (69.8)
Negative	40/202 (19.8)
Unknown	21/202 (10.4)
Homeless	
Yes	35/202 (17.3)
No	165/202 (81.7)
Unknown	2/202 (0.99)
Preexisting heart disease	
Congenital	2/202 (0.99)
Intracardiac device	1/202 (0.49)
Unknown	12/202 (5.9)
Substance use	
Opioid	19/202 (9.4)
Stimulant	46/202 (22.8)
Polysubstance	113/202 (55.9)
Unknown	24/202 (11.9)
Health care–associated IE	0
Site of IE	
Right side	125/202 (61.8)
Left side	55/202 (27.2)
Bilateral	13/202 (6.4)
Unknown^a^	9/202 (4.5)
Prosthetic valve	2/202 (1.0)
Primary valve	
Aortic	26/202 (12.9)
Mitral	24/202 (11.9)
Tricuspid	118/202 (58.4)
Pulmonic	2/202 (1.0)
>1 Structure involved	20/202 (9.9)
Unknown^a^	9/202 (4.5)
Organism	
Methicillin-sensitive *Staphylococcus aureus*	113/202 (55.9)
Methicillin-resistant *Staphylococcus aureus*	43/202 (21.3)
Coagulase-negative staphylococci	1/202 (0.49)
Viridans group streptococci	4/202 (1.9)
Streptococci (non–viridans group)	5/202 (2.5)
Enterococci	11/202 (5.4)
Enterobacteriaceae	1/202 (0.5)
HACEK	0
*Pseudomonas* or *Acinetobacter*	2/202 (1.0)
Fungal	1/202 (0.5)
Polymicrobial	13/202 (6.4)
Culture negative	5/202 (2.5)
*Granulicatella adiacens*	1/202 (0.5)
Recurrent IE	
Total No. of patients	59/202 (29.2)
1 Recurrent episode	32/202 (15.8)
>1 Recurrent episode	27/202 (13.4)
Unknown	2/202 (1.0)
Relapsed IE	
1 Relapse	34/202 (17.3)
>1 Relapse	17/202 (8.4)
Unknown	2/202 (1.0)
Invasive infection	
Osteomyelitis	13/202 (6.4)
Septic arthritis	23/202 (11.4)
Central nervous system infection^b^	13/202 (6.4)
Cardiac complications	
Myocardial or aortic root abscess	12/202 (5.9)
Unknown	12/202 (5.9)
Congestive heart failure	30/202 (14.9)
Unknown	16/202 (7.9)
Conduction delay	4/202 (1.9)
Vascular complications	
Ischemic stroke	37/202 (18.3)
Intracerebral hemorrhage	18/202 (8.9)
Mycotic aneurysm	7/202 (3.5)
Septic pulmonary emboli	129/202 (63.9)
Secondary bacteremia	41/202 (20.3)
Length of stay, median (IQR), d	21.5 (12.7-43)
Left against medical advice	34/202 (16.8)
Treatment route	
Intramuscular	2/202 (0.99)
Intravenous	162/202 (80.2)
Oral	38/202 (18.9)
Peripherally inserted central catheter line insertion	
Yes	172/202 (85.1)
No	24/202 (11.9)
Unknown	6/202 (2.9)
Peripherally inserted central catheter line abuse	42/202 (20.8)
Surgical treatment	39/202 (19.3)
Surgical procedure	
Device insertion or removal	2/39(5.1)
Valve repair	18/39 (46.2)
Valve replacement	17/39 (43.6)
Valve repair and replacement	2/39 (5.1)
Opioid substitution therapy	34/202 (16.8)
Referral to addiction treatment	40/202 (19.8)
Death	68/202 (33.7)

Abbreviations: HACEK, *Haemophilus* species, *Aggregatibacter* species, *Cardiobacterium hominis*, *Eikenella corrodens*, and *Kingella* species; IE, infective endocarditis; IQR, interquartile range.

^a^ Unknown indicates that endocarditis diagnosis was based on 1 major and 3 or more minor modified Duke criteria in the absence of a definite vegetation on echocardiogram.

^b^ Central nervous system infection includes meningitis, epidural abscess, paraspinal abscess, and brain abscess.

Surgical treatment was undertaken in 19.3% of cases (39 of 202). As shown in Table 2, most surgically treated patients had left-sided infections (56.4% [22 of 39]), and valve repair and replacement were performed with similar frequency (in 18 and 17 patients, respectively). There was no difference between age or comorbidities of surgically treated patients when compared with medically treated patients. Patients referred to addiction treatment were not more likely to have surgery (10 of 39 [25.6%] vs 30 of 163 [18.4%]; risk ratio [RR], 1.39; 95% CI, 0.75-2.61; *P* = .43). Furthermore, there was no difference in the proportion of surgically treated patients vs medically treated patients discharged with OST (5 of 39 [12.8%] vs 29 of 163 [17.8%]; RR, 0.72; 95% CI, 0.29-1.75; *P* = .63). Appropriately, patients with an indication for surgery, specifically a myocardial or aortic root abscess, congestive heart failure, or conduction delay, were more likely to be treated surgically; however, a proportion of these patients were also exclusively treated medically. For medical management, a PICC line was used in most cases (172 of 202 [85.1%]). Misuse of these lines was suspected by the clinical team in one-fifth of PWID (42 of 202 [20.8%]), leading to secondary bacteremia in 41 patients. Rates of invasive infections and vascular complications were not significantly different, apart from septic pulmonary emboli occurring more frequently (43.6% [17 of 39] vs 66.7% [112 of 163]; RR, 0.63; 95% CI, 0.44-0.92; *P* = .006) in the medically managed group (likely reflecting patients with tricuspid valve disease). Length of hospital stay did not differ between groups, although patients who underwent surgery had a higher rate of preoperative ICU admission (58.9% [23 of 39] vs 32.7% [55 of 163]; RR, 1.75; 95% CI, 1.25-2.45; *P* = .006).

**Table 2.** Clinical Characteristics of Persons Who Inject Drugs Treated Surgically vs Medically

Variable	No./Total No. (%)		*P* Value	RR (95% CI)
	Surgical (n = 39)	Medical (n = 163)		
Age, median (IQR), y	37 (28.5-42)	34 (28-42)	.30	
Sex				
Male	24/39 (61.5)	80/163 (49.1)	.13	1.31 (0.98-1.73)
Female	14/39 (35.9)	83/163 (50.9)		0.71 (0.45-1.10)
HIV positive	3/39 (7.7)	13/163 (7.9)	>.99	0.80 (0.24-2.66)
Hepatitis C positive	28/39 (71.8)	113/163 (69.3)	.82	0.96 (0.79-1.18)
Homeless	5/39 (12.8)	30/163 (18.4)	.49	0.69 (0.29-1.66)
Preexisting heart disease				
Congenital	1/39 (2.6)	1/163 (0.61)	.35	4.18 (0.27-65.4)
Intracardiac device	1/39 (2.6)	0	.20	
Site of IE				
Right side	12/39 (30.8)	113/163 (69.3)	<.001	0.44 (0.27-0.72)
Left side	22/39 (56.4)	33/163 (20.2)		2.84 (1.87-4.29)
Bilateral	5/39 (12.8)	8/163 (4.9)		2.61 (0.91-7.55)
Prosthetic valve	2/39 (5.1)	0	.04	
Primary valve				
Aortic	10/39 (25.6)	16/163 (9.8)	.02	2.61 (1.28-5.31)
Mitral	9/39 (23.1)	15/163 (9.2)	.03	2.51 (1.19-5.31)
Tricuspid	12/39 (30.8)	106/163 (65.1)	<.001	0.47 (0.29-0.76)
Pulmonic	0	2/163 (1.2)	>.99	
>1 Structure involved	8/39 (20.5)	12/163 (7.4)	.03	2.79 (1.22-6.35)
Organism				
Methicillin-sensitive *Staphylococcus aureus*	20/39 (51.3)	93/163 (57.1)	<.001	0.89 (0.64-1.25)
Methicillin-resistant *Staphylococcus aureus*	3/39 (7.7)	40/163 (24.5)		0.31 (0.10-0.96)
Coagulase-negative staphylococci	1/39 (2.6)	0		
Viridans group streptococci	2/39 (5.1)	2/163 (1.2)		4.17 (0.61-28.7)
Streptococci (non–viridans group)	2/39 (5.1)	3/163 (1.8)		2.79 (0.48-16.1)
Enterococci	7/39 (17.9)	4/163 (2.5)		7.31 (2.25-23.7)
Enterobacteriaceae	1/39 (2.6)	0		
*Pseudomonas* or *Acinetobacter*	1/39 (2.6)	1/163 (0.61)		4.18 (0.27-65.4)
Fungal^a^	0	1/163 (0.61)		
Polymicrobial	1/39 (2.6)	13/163 (7.9)		0.35 (0.05-2.59)
Culture negative	0	5/163 (3.1)		
*Granulicatella adiacens*	0	1/163 (0.61)		
Recurrent IE				
No recurrence	26/39 (66.7)	115/163 (70.6)	.69	0.93 (0.73-1.19)
1 Recurrent episode	9/39 (23.1)	23/163 (14.1)		1.61 (0.81-3.21)
>1 Recurrent episode	4/39 (10.3)	23/163 (14.1)		0.72 (0.26-1.96)
Invasive infection				
Osteomyelitis	2/39 (5.1)	11/163 (6.7)	>.99	0.76 (0.18-3.29)
Central nervous system infection	2/39 (5.1)	11/163 (6.7)	>.99	1.02 (0.94-1.11)
Septic arthritis	3/39 (7.7)	20/163 (12.3)	.06	0.63 (0.19-2.01)
Cardiac complications^a^				
Myocardial or aortic root abscess	9/39 (23.1)	3/163 (1.8)	<.001	12 (3.41-42.2)
Congestive heart failure	10/39 (25.6)	20/163 (12.3)	.04	2.08 (1.07-4.06)
Conduction delay	4/39 (10.3)	0	.001	
Vascular complications				
Ischemic stroke	11/39 (28.2)	26/163 (15.9)	.12	1.77 (0.96-3.26)
Intracerebral hemorrhage	6/39 (15.4)	33/163 (20.2)	.12	2.09 (0.84-5.22)
Mycotic aneurysm	0	7/163 (4.3)	.35	
Septic pulmonary emboli	17/39 (43.6)	112/163 (68.7)	.006	0.63 (0.44-0.92)
Length of stay, median (IQR), d	24 (16.5-44)	21 (12-43)	.25	
Intensive care unit admission	23/39 (58.9)	55/163 (33.7)	.006	1.75 (1.25-2.45)
Septic shock	19/39 (48.7)	57/163 (34.9)	.16	1.39 (0.95-2.05)
Left against medical advice	2/39 (5.1)	32/163 (19.6)	.03	0.26 (0.07-1.04)
Treatment route				
Intramuscular	0	2/163 (1.2)	.26	
Intravenous	35/39 (89.7)	127/163 (77.9)		1.15 (1.01-1.32)
Oral	4/39 (10.3)	34/163 (20.9)		0.49 (0.19-1.31)
Peripherally inserted central catheter line				
Insertion	36/39 (92.3)	136/163 (83.4)	.05	1.14 (1.05-1.24)
Abuse	4/39 (10.3)	38/163 (23.3)	.08	0.44 (0.17-1.16)
Opioid substitution therapy	5/39 (12.8)	29/163 (17.8)	.63	0.81 (0.34-1.93)
Referral for addiction treatment	10/39 (25.6)	30/163 (18.4)	.43	1.39 (0.75-2.60)

Abbreviations: IE, infective endocarditis; IQR, interquartile range; RR, relative risk.

^a^ Indication for surgery.

In total, there were 68 deaths (33.7% mortality rate). Cause of death is shown in eTable 1 in the Supplement, where the majority were secondary to sepsis (49 of 68 [72.1%]). Survival curves are shown in the Figure. The survival of all PWID with first-episode endocarditis is shown in panel A, and the remainder of the curves illustrate survival when stratified by surgical treatment at 30 days, 6 months, and 1 year. Survival was not significantly different at any time point. To further explore surgical management in these patients, additional survival analysis was undertaken. Variables identified as having an association with mortality among all patients included age, site of infection, leaving AMA, and referral to addiction treatment (Table 3), where a higher risk was seen only with the site of infection for left-sided endocarditis (RR, 1.98; 95% CI, 1.28-3.08) and bilateral infection (RR, 3.18; 95% CI, 1.08-9.31). Reduced mortality was associated with leaving AMA and referral to addiction treatment. Adjusting for age and sex, multivariable analysis identified a significantly lower mortality associated with surgery (HR, 0.44; 95% CI, 0.23-0.84; *P* = .01) and referral to addiction treatment (HR, 0.29; 95% CI, 0.12-0.73; *P* = .008) (Table 4). Conversely, worse outcomes were associated with left-sided infection (HR, 3.26; 95% CI, 1.82-5.84; *P* < .001) and bilateral involvement (HR, 4.51; 95% CI, 2.01-10.1; *P* < .001). Sensitivity analysis (eTable 2 in the Supplement) illustrates the association of reduced mortality with surgery unless valvular involvement is removed from the model.

**Figure.**
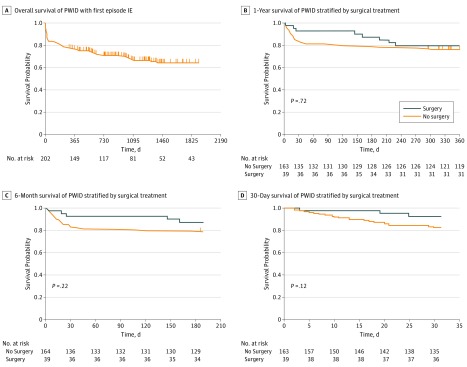
Survival Curves for Persons Who Inject Drugs (PWID) With First-Episode Infective Endocarditis (IE) Survival curves are shown for PWID with IE who underwent surgery vs those who did not. Survival was not significantly different at any time point.

**Table 3.** Variables Associated With All-Cause Mortality in First-Episode Infective Endocarditis Among Persons Who Inject Drugs

Variable	Mortality, No. (%) (n = 68)	*P* Value	RR (95% CI)
Age at death, median (IQR), y	36.5 (29-44.25)	.04	
Sex			
Male	41 (60.3)	.12	1.26 (0.97-1.64)
Female	27 (39.7)		0.76 (0.54-1.06)
Surgery			
Yes	13 (19.1)	>.99	0.98 (0.54-1.79)
No	55 (80.9)		1.01 (0.87-1.16)
Causative organism			
*Staphylococcus aureus*^a^	48 (70.6)	.27	0.88 (0.74-1.04)
Polymicrobial	6 (8.8)		1.69 (0.59-4.83)
Other	14 (20.6)		1.24 (0.64-2.41)
Site of IE			
Right side	30 (44.1)	<.001	0.61 (0.45-0.81)
Left side	28 (41.2)		1.98 (1.28-3.08)
Bilateral	8 (11.8)		3.18 (1.08-9.31)
Left against medical advice	5 (7.4)	.01	0.34 (0.14-0.84)
Substance use			
Opioid	20 (29.4)	.11	1.72 (1.06-2.80)
Stimulant	5 (7.4)		0.79 (0.30-2.11)
Polysubstance	30 (44.1)		0.81 (0.62-1.06)
Opioid substitution therapy	9 (13.2)	.43	0.34 (0.43-1.33)
Referral to addiction treatment	5 (7.4)	.001	0.28 (0.12-0.69)

Abbreviations: IE, infective endocarditis; IQR, interquartile range; RR, relative risk.

^a^ Includes methicillin-sensitive and methicillin-resistant *Staphylococcus aureus*.

**Table 4.** Unadjusted and Adjusted Cox Proportional Hazards Model for Mortality in First-Episode Infective Endocarditis in Persons Who Inject Drugs

Variable	Unadjusted		Adjusted	
	HR (95% CI)	*P* Value	HR (95% CI)^a^	*P* Value
Treatment				
Medical	1 [Reference]		1 [Reference]	
Surgical	0.89 (0.49-1.64)	.72	0.44 (0.23-0.84)	.01
Site of infection				
Right side	1 [Reference]		1 [Reference]	
Left side	2.92 (1.76-4.87)	<.001	3.26 (1.82-5.84)	<.001
Bilateral	3.96 (1.80-8.68)	<.001	4.51 (2.01-10.1)	<.001
Left against medical advice				
No	1 [Reference]		1 [Reference]	
Yes	0.34 (0.14-0.85)	.02	0.47 (0.18-1.19)	.11
Referral to addiction treatment				
No	1 [Reference]		1 [Reference]	
Yes	0.28 (0.11-0.69)	.006	0.29 (0.12-0.73)	.008

Abbreviation: HR, hazard ratio.

^a^ Adjusted for age and sex.

## Discussion

To our knowledge, this is one of the largest, most contemporary cohorts of PWID with IE. Our data are in keeping with previous studies showing that PWID with IE have predominantly right-sided disease caused by *S aureus* and high mortality. We aimed to characterize surgical cases and provide further observational data regarding whether certain patients may benefit from surgical treatment. We also focused our analysis on the impact of referral to addiction services on survival. Optimization of treatment strategies in this population is important given the increasing frequency of injection drug use,^18,19,20^ hospitalizations related to injection drug use,^8,21,22^ and injection drug use–associated IE.^7,12^

Infective endocarditis associated with injection drug use is hypothesized to be secondary to repeated injection of particulate matter, use of unsterile injection technique introducing skin flora into the bloodstream, contamination of injection equipment by saliva or unsterile water,^22,23,24,25^ or extension from skin and soft-tissue infections.^11,23,24,25^ The increase in IE is felt to represent an increasing number of PWID, increasing injection frequency,^25^ or use of drugs that may have a higher incidence of IE.^26^ Hepatitis C infection has been proposed as a surrogate to identify PWID given the concurrent increase in cases and related hospitalizations.^20,22,27^ In our cohort, the majority of PWID were positive for hepatitis C (69.8%). Our finding that *S aureus* is the most common causative organism is consistent with previous results.^2,21,23,28,29,30,31^ The high percentage of *S aureus* in patients who inject drugs with first-episode IE (77.2% [156 of 202]) likely reflects the ability of these organisms to infect native healthy valves, whereas non–drug users more commonly have preexisting congenital heart disease, degenerative valvular disease, or intracardiac devices that can be infected by lower-virulence organisms such as coagulase-negative staphylococci, viridans group streptococci, and enterococci. Among our cohort, PWID had a higher rate of right-sided infections (61.4% [124 of 202]), consistent with the right-sided predominance that has been reported previously.^23,24,26,28^

Overall, factors associated with mortality in PWID populations have not been well described. Univariate analysis suggested age, site of infection, leaving AMA, and referral to addiction treatment as variables that may affect mortality. On subsequent multivariable analysis, we identified left-sided infection and bilateral infection as having a higher HR, in keeping with previous reports that both PWID and non–drug users with right-sided disease have a more favorable prognosis^27,32^ (Table 4). Regression analysis did not demonstrate an association between the causative organism and mortality; some studies^33,34,35,36^ have suggested that *S aureus* IE confers a poorer prognosis, while others have not.^23^

Surgery has repeatedly been shown to improve outcomes in IE in non–drug users.^34,35,37,38,39,40^ It is suggested that endocarditis caused by *S aureus* and other virulent microorganisms should be managed surgically during initial hospitalization.^41^ Current guidelines also recommend early surgery once an indication has been established.^42^ Operative management for IE remains controversial in PWID as there is a significant risk of requiring reoperation due to recurrent injection drug use and recurrent IE^43,44,45^ or reinfection of a prosthesis^28^ (particularly as prosthetic valve IE is associated with higher mortality).^2,21^ The literature focusing on surgical management in PWID populations is hindered by small sample sizes, potentially reflecting the bias toward medical management in these patients. Additionally, it is possible that differences in long-term survival between patients who inject drugs and those who do not are not fully dependent on reoperation alone.^45^

Previous studies assessing the potential benefit of surgical therapy in PWID have primarily assessed the outcomes of PWID vs non–drug users who were all treated surgically and have found a higher need for reoperation, particularly due to reinfection, but not generally a higher death rate in patients who inject drugs.^20,36,45,46,47,48^ These studies were limited by the small sample sizes of PWID and markedly different ages and comorbidities^20,49^ of the 2 populations, which limits the conclusions that could be drawn. Notably, it has also been shown that PWID selected for surgery were more urgent cases (taken to the operating room within 24 hours) and more likely to have active infection, with the causative agent being *S aureus,*^50^ suggesting a more unstable population. This may contribute to some of the differences in outcomes seen in the early postoperative period, specifically the need for reoperation or early mortality.^48^

In contrast to previous studies, our analysis of a large cohort of PWID with IE allowed assessment of the association between surgery and survival by comparing PWID treated surgically vs nonsurgically. As demonstrated by the survival curves, which show no significant difference in survival between patients treated surgically and those treated medically, the potential benefit of surgery is influenced by other clinical variables. Multivariate analysis and subsequent sensitivity analysis suggest that the site of infection is an important factor. It is possible surgery was not associated with mortality on the unadjusted analysis because it was often done on patients who had a worse prognosis due to left-sided or bilateral disease. Patients who underwent surgery also may have been more ill, as they were more likely to have been managed preoperatively in the ICU and to have congestive heart failure, an aortic root abscess or myocardial abscess, or conduction delay than patients who did not have surgery. When controlling for additional characteristics, surgery was associated with significantly lower mortality. Nevertheless, we cannot rule out the presence of other unmeasured confounders, so further study will be necessary to identify optimal indications for surgery in PWID. It is notable that presently the American Society for Thoracic Surgery consensus guidelines^42^ recommend using the same criteria for surgery in patients who inject drugs and those who do not. An optimal approach to surgical treatment of PWID involves a multidisciplinary team, in which involvement of ethics or patient commitment to rehabilitation prior to operation should be considered part of a complete treatment plan.^33^

Harm reduction strategies are not widely used in hospital settings,^51^ and hospitalization represents a meaningful opportunity to engage PWID.^52^ However, it has been shown that addiction interventions are often suboptimal among PWID with IE,^53^ and among our cohort the referral rate for addiction treatment was 19.8% (40 of 202). A similar percentage of patients were discharged with OST (such as buprenorphine or methadone). Use of OST has been demonstrated to reduce mortality and increase the chance of long-term cessation of injection drug use.^54^ Our findings support the recent recommendations from the National Academies of Sciences, Engineering, and Medicine that emphasize the importance of integrating treatment for opioid use disorder with acute care for infectious diseases.^55^

### Limitations

Our analysis is inherently limited because it is retrospective in nature. Our results are limited to patients who fulfilled the modified Duke criteria for a diagnosis of definite endocarditis. Patients with possible endocarditis were excluded; therefore, our results cannot be generalized to this population of patients. Unfortunately, data regarding the specifics of medical treatment following discharge (agent, duration, and completion) were not collected, which could affect presentation with an indication for surgical treatment. Home-based intravenous treatment was provided in the community by an outpatient nursing program; therefore, home care notes regarding treatment adherence and completion were unavailable. Incomplete treatment would likely affect survival, especially among PWID, for whom fractured health care contact is common; however, our results did not reflect decreased survival among PWID who left AMA. Interestingly, leaving AMA was associated with lower mortality on univariate analysis. However, in the adjusted multivariable analysis, the finding of lower mortality associated with leaving AMA was no longer seen. We suspect that patients who were less ill (eg, those without left-sided or bilateral disease, embolic disease, or metastatic infections) were more likely to be well enough to sign out AMA. Recent data suggesting that partial oral regimens may be effective in infectious endocarditis^56^ suggest that patients who left AMA with prescriptions for oral therapy may have done well. We cannot rule out the possibility that the patients selected for surgery were felt to have less severe addiction issues and therefore were a select group with better addiction prognosis, although it is important to note the association with lower mortality identified in the multivariable model. Surgery was associated with lower mortality in multivariable models that included referral to addictions services and discharge with OST. Although surgery was associated with a reduction in mortality, we cannot rule out that unmeasured variables (such as a clinical impression of low risk for relapse of drug use) led to selection of patients with improved prognosis for surgery. Additionally, owing to sample size, it was not possible to assess the impact of valve repair vs valve replacement; this is significant when considering surgery in PWID because of the risk of reinfection of a prosthetic valve. It is likely that reinfection of a repaired valve may not have the same grave prognosis as prosthetic valve endocarditis. Further research is necessary to determine the optimal candidates for surgical management in PWID and should also explore increased use of addiction treatment.

## Conclusions

We describe PWID with first-episode IE and highlight the current epidemiology and management of these patients. We highlight the potentially important role of referral to addiction services. Further study to identify PWID who would benefit from surgery is warranted.

## Notes

Supplement. **eTable 1.** Cause of Death in PWIDs With First Episode Infective Endocarditis **eTable 2.** Sensitivity Analysis Using Cox Proportional Hazard Model for All-Cause Mortality in First Episode Infective Endocarditis Focused on Surgical Treatment
