# Supplementary material for: Clinical Characteristics and Factors Associated With Mortality in First-Episode Infective Endocarditis Among Persons Who Inject Drugs
Source: JAMA Netw Open. 2018 Nov 21;1(7):e185220. doi: 10.1001/jamanetworkopen.2018.5220 (PMC6324402; doi:10.1001/jamanetworkopen.2018.5220)
Supplement: Supplement. — eTable 1. Cause of Death in PWIDs With First Episode Infective Endocarditis eTable 2. Sensitivity Analysis Using Cox Proportional Hazard Model for All-Cause Mortality in First Episode Infective Endocarditis Focused on Surgical Treatment [file jamanetwopen-1-e185220-s001.pdf]

Supplementary Online Content

Rodger L, Glockler-Lauf SD, Shojaei E, et al. Clinical characteristics and factors associated with mortality in first-episode infective endocarditis among persons who inject drugs. *JAMA Netw Open*. 2018;1(7):e185220. doi:10.1001/jamanetworkopen.2018.5220

eTable 1. Cause of Death in PWIDs With First Episode Infective Endocarditis

eTable 2. Sensitivity Analysis Using Cox Proportional Hazard Model for All-Cause Mortality in First Episode Infective Endocarditis Focused on Surgical Treatment

This supplementary material has been provided by the authors to give readers additional information about their work.

© 2018 Rodger L et al. *JAMA Network Open.*

eTable 1: Cause of Death in PWIDs with First Episode Infective Endocarditis	
**Cause of Death**	**Patients (n=68, No., %)**
Non-Endocarditis Related	
Status Asthmaticus	1 (1.5%)
Trauma	1 (1.5%)
Endocarditis Related	
Sepsis*	49 (72.1%)
Heart Failure	2 (2.9%)
Conduction Disease or Arrhythmia**	2 (2.9%)
Pulmonary Hemorrhage or Embolism	2 (2.9%)
Intracerebral Hemorrhage	3 (4.4%)
Embolic Stroke	7 (10.3%)
Pneumothorax***	1 (1.5%)

*includes patients with sepsis and an additional listed cause of death (cerebrovascular event, congestive heart failure)
**includes complete heart block, ventricular tachycardia
***pneumothorax secondary to septic emboli rupture

© 2018 Rodger L et al. *JAMA Network Open.*

eTable 2: Sensitivity Analysis Using Cox Proportional Hazard Model for All-Cause Mortality in First Episode Infective Endocarditis Focused on Surgical Treatment		
**Model**	**HR for Surgery (95% CI)**	**p-value**
*Primary analysis**	0.44 (0.23 – 0.84)	0.01
Excluding AMA	0.44 (0.23 – 0.84)	0.01
Excluding Referral	0.44 (0.23 – 0.84)	0.01
Excluding Site of Infection	0.75 (0.44 – 1.26)	0.35
Including OST	0.43 (0.23 – 0.83)	0.01
*refers to final model as shown in Table 4		

© 2018 Rodger L et al. *JAMA Network Open.*
